# The effect of virtual reality on reducing patients’ anxiety and pain during dental implant surgery

**DOI:** 10.1186/s12903-024-03904-8

**Published:** 2024-02-05

**Authors:** Alireza Ghobadi, Hedaiat Moradpoor, Hamid Sharini, Habibolah Khazaie, Pooya Moradpoor

**Affiliations:** 1 Students Research Committee, School of Dentistry, Kermanshah University of Medical Sciences, Kermanshah, Iran; 2 Department of Prosthodontics, School of Dentistry, Kermanshah University of Medical Sciences, Kermanshah, Iran; 3 Department of Biomedical Engineering, Faculty of Medicine, Kermanshah University of Medical Sciences (KUMS), Kermanshah, Iran; 4 Sleep Disorders Research Center, Kermanshah University of Medical Sciences (KUMS), Kermanshah, Iran; 5 Department of business management, Central Tehran branch, Islamic Azad University, Tehran, Iran

**Keywords:** Virtual reality, Anxiety, Pain, Implant, Biofeedback, Dentistry

## Abstract

**Background:**

Dental anxiety and pain pose serious problems for both patients and dentists. One of the most stressful and frightening dental procedures for patients is dental implant surgery; that even hearing its name causes them stress. Virtual reality (VR) distraction is an effective intervention used by healthcare professionals to help patients cope with unpleasant procedures. Our aim is to evaluate the use of high-quality VR and natural environments on dental implant patients to determine the effect on reducing pain and anxiety.

**Methods:**

Seventy-three patients having two dental implant surgeries participated in a randomized controlled trial. One surgery was with VR, and one was without. Anxiety was measured with the the State-Trait Anxiety Inventory and the Modified Dental Anxiety Scale tests. The pain was measured with the Numerical Rating Scales. Patient satisfaction, surgeon distress, memory vividness, and time perception were evaluated. Physiological data were collected with biofeedback and neurofeedback device.

**Results:**

VR effectively reduced anxiety and pain compared to no VR. Physiological data validated the questionnaire results. Patient satisfaction increased, with 90.4% willing to reuse VR. VR reduced time perception and memory vividness.

**Conclusion:**

Psychometric and psychophysiological assessments showed that VR successfully reduced patient pain and anxiety. More dental clinicians should use VR technology to manage patient anxiety and pain.

## Introduction

The history of dentistry is almost as long as the history of human civilization [15], and one of the most challenging aspects of dental care that dentists face today is managing patients’ pain and anxiety [5]. Despite advances in dental technologies and treatments, many people still avoid or delay dental treatments due to high levels of fear of pain and anxiety [29].

*Anxiety* is a common response to surgery [40], especially since being awake during surgery with the help of local anesthetics can be full of specific fears and anxieties [16, 34, 51]. Not only is anxiety unpleasant, but a consistent relationship has been observed between surgical anxiety, postoperative pain [4, 18], an increased need for analgesics [41], and delayed recovery [35]. Results from a large adult dental health survey by the National Health Service (NHS) showed that over one-third of adults (36%) suffer from moderate dental anxiety, and a further 12% suffer from extreme dental anxiety. These patients only go to the dentist when experiencing pain, thus exacerbating their anxiety too [3]. In addition, anxious patients are less interested in keeping their routine appointments [24], their treatment takes longer, they feel less satisfied after treatment [28], and the dentists themselves experience more distress when treating anxious patients [12].

*Pain* is a complex, multidimensional subjective experience involving sensory, emotional, and cognitive processes. Pain perception is influenced by a wide spectrum of dimensions and interactions between these processes, with the cognitive-evaluative, motivational-affective, and discriminative dimensions all playing a role in pain perception [36, 37]. Discomfort from Pain is frequently reported by patients undergoing dental treatment, even during routine restorative procedures [26, 27]. In one population-based study, 71% of respondents reported a negative dental experience related to pain, with 30% reporting three or more painful incidents [30]. This is compared to lifetime prevalence data, where 60% of respondents stated their last dental visit was painful [50]. Additional evidence comes from a longitudinal study in which 40% of respondents over a 5-year period reported experiencing painful dental treatment [32]. Painful treatment episodes lead to the development of dental fear and irregularities in routine visits [30, 45]. Reporting previous painful treatment episodes predicts pain during subsequent dental procedures over a 5-year follow-up [32]. Thus, “pain begets pain,” and a vicious cycle develops associated with postponing dental visits. Irregular visits prevent the treatment of minor issues, leading to the use of more stressful dental procedures and increasing the chances of further pain provocation [23].

In recent years, 19% of the population above age 35 has undergone dental implant treatment. While implant placement is a relatively simple surgical procedure for the surgeon, it is usually associated with a high level of anxiety and discomfort for the patient. Even hearing the words “implant surgery” increases anxiety levels for many patients [9]. In the past, the major focus for managing patient pain and anxiety was on pharmacological interventions, while articles published in the past decade have increasingly focused on non-pharmacological techniques. One such strategy is distraction, a technique based on the concept of limited human capacity for attention. Distraction techniques range from passive to active interventions, with the belief that the more interactive the distraction technique, the greater the potential for diversion from pain, as some level of attention is required to experience pain [25, 44].

*Virtual reality (VR)* is an advanced technology system that allows users to become fully immersed in a multisensory (e.g., visual, auditory and haptic) “virtual world” experience. VR is rapidly emerging in popular culture and has gained popularity as an innovative distraction technique in recent years. The cost of a VR system has dramatically decreased since the mid-1990s, while the quality, portability, and technology have markedly improved. VR’s immersive and interactive aspects compete for patients’ attention, thus minimizing patients’ ability to process incoming signals. This highly immersive and multisensory VR experience is distinct from common forms of distraction (e.g., bubbles, books, toys), passive TV or movie watching, or a computer/gaming console video game. For example, Kipping et al. [22] showed that VR distraction resulted in less analgesic medication being used compared to standard distractions like TV, music, or stories. The benefits of using VR to reduce dental anxiety and pain during dental procedures have been extensively addressed in the scientific literature, and its utility as a distraction tool has garnered increasing attention in medical fields. Earlier versions of VR have been evaluated as a means to reduce perceived pain, anxiety, and general distress during painful medical procedures such as wound care, chemotherapy, surgery, physiotherapy, dental procedures, and general medicine [8, 31]. With the increasing use of VR as a distraction intervention in healthcare settings, it is important to address how successful VR interventions are in helping dental implant candidates cope with pain and anxiety. Therefore, we tested the following three hypotheses:

First, we predicted VR would decrease patients’ overall pain and anxiety during dental implant surgery.

Second, we proposed that, in line with Elaborated Intrusions (EI) theory, providing VR distraction would result in less vivid memories one week later.

Third, based on previous research showing VR can influence time perception, we hypothesized that providing VR distraction during dental treatment would result in lower time perception compared to no VR distraction [43].

## Material & methods

### Regulatory approvals

Before participating in the study, each patient was given detailed information about the study. The procedures performed in this study were in accordance with the Helsinki Declaration of 1964 and approved by the Ethics Committee of Human Research at Kermanshah University of Medical Sciences (IR.KUMS.REC.1402.076) with the clinical trial code IRCT20230612058461N1 (20/06/2023).

### Study design

**Fig. 1:**
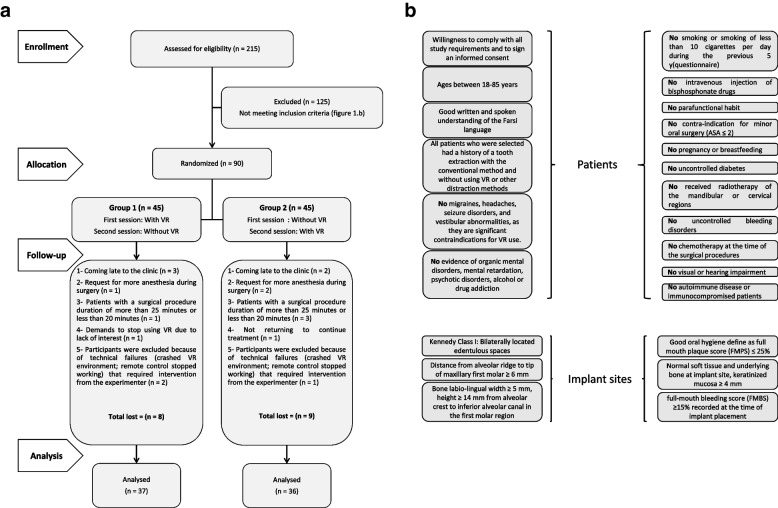
a: CONSORT diagram showing trial enrolment, allocation, follow-up and analysis. b: Inclusion Criteria

A randomized, controlled crossover clinical trial with two intervention groups was conducted. After screening and completing patient records, obtaining necessary consent, and presenting the treatment plan, patients were scheduled for implant surgery on the lower right and left first molars. A blinded third-party statistician then used computer software (List Randomizer, Waterloo, Ireland) to assign identification numbers to two groups randomly. This information was concealed in sealed envelopes and opened on the day of surgery. This clinical trial was conducted over two sessions for each patient (Fig. 1). In group 1, patients underwent implant surgery on the first lower molar tooth using VR. In the second session, they underwent implant surgery on the other first lower molar tooth without VR. In group 2, patients first underwent implant surgery on the first lower molar tooth without VR. In the next session, they underwent implant surgery on the other first lower molar tooth using VR.

**Fig. 2:**
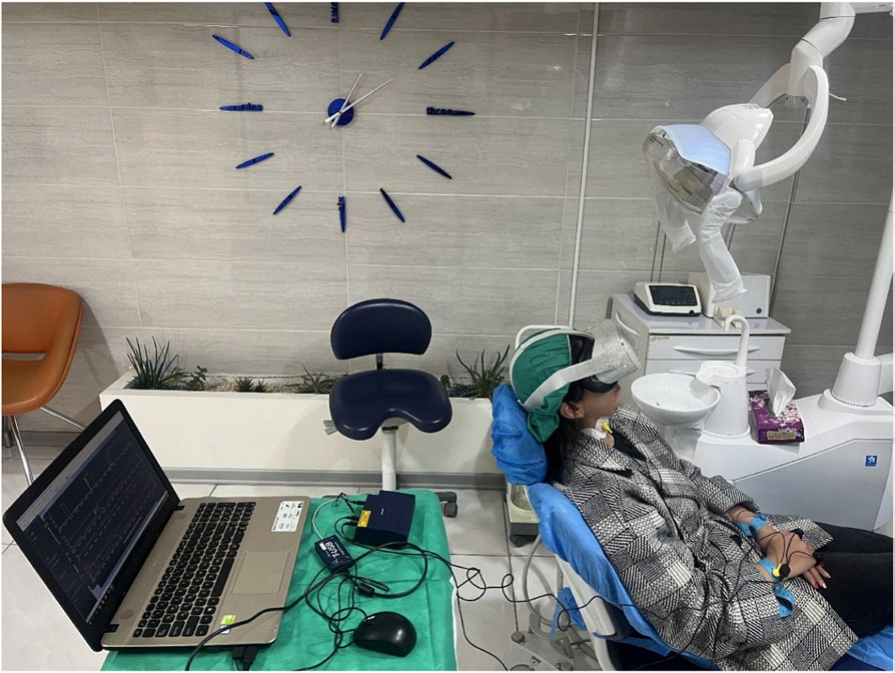
Patient and sensors preparations. A large clock was installed in front of the surgeon to keep track of the time

**Fig. 3:**
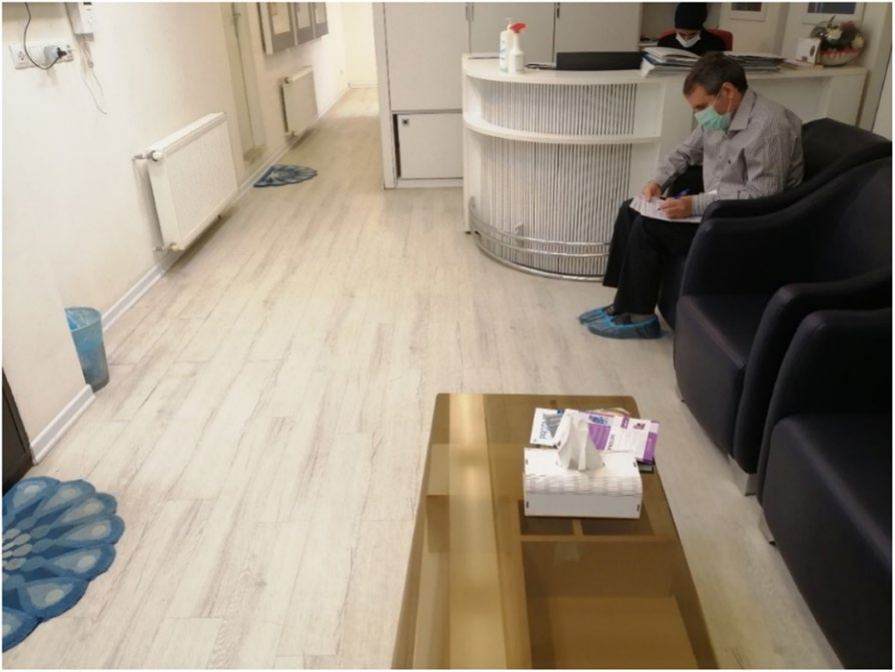
The patient’s waiting room was quiet and no music was played. The waiting time for each patient was a maximum of 10 minutes

Prior to the study, the authors’ preliminary examinations of 10 patients showed that the average length of the implant surgery procedure by the surgeon in this study was 23 minutes (without considering patient preparation time and anesthetic injection). To maximize the standardization of conditions for all patients, those whose surgery length was over 25 minutes or less than 20 minutes were excluded from the study. For those patients whose surgical process was between 20 to 25 minutes, the surgeon used other dental tools and pretended to work, trying to increase the treatment duration to 25 minutes so that the actual time spent was the same for all patients. For this purpose, a large clock was installed in front of the surgeon to keep track of time (Fig. 2). To maximize standardization of conditions, all patients were asked to attend the office alone. When in the waiting room, no other patients were present, and silence prevailed (Fig. 3). The pens used were all new and of the same brand to minimize the effect of other experimental interventions.

### Participants

Eligible patients for this study were invited from a university of medical sciences and several private clinics to the prosthodontist specialist’s office (Hedaiat Moradpoor). No discrimination was made based on race, gender, or socioeconomic status. Patients received a 10% discount on treatment if they participated in this study. The sample size was calculated using the results of previous studies. In the study by Tanja-Dijkstra et al. [48], the standard deviation of the Experienced pain variable was *σ*_*w*_ = 1.95.. The mean of the Active VR and control groups was ${\overline{X}}_T$ = 2.15 and ${\overline{X}}_R$ = 1.72, respectively. With α = 0.05 and power of 1 - β = 90%, the minimum sample size was 70. The sample size was calculated using PASS software. For this purpose, 215 patients were examined, 90 of whom met the eligibility criteria to participate in the study. 17 people dropped out during the study, and 73 patients (including 36 men and 37 women) with a mean age of 44.29 ± 12.98 years consented to participate in this study.

### Hardware

#### VR headset

The Oculus Quest 2 virtual reality headset from Meta was purchased and used for this study. This headset consists of a display with a resolution of 3664 × 1920 pixels with a 100-degree field of view, a refresh rate of 90 Hz, and a weight of 830 g. This headset can be placed on glasses. The reasons for choosing this headset for this study include being wireless, easy to use for watching movies, and having external speakers so the patient can hear the movie music and the surgeon’s instructions clearly and follow them.

#### Biofeedback & neurofeedback system

During each treatment session, psychophysiological parameters (electromyography (EMG), electrocardiography (EKG), and skin conductance response (SCR)) were recorded to obtain objective criteria for patients’ emotional states. Data was recorded using the FlexComp Infiniti - 10 Channel System (Thought Technology Ltd., Montreal, Quebec, Canada). This 10-channel polygraph and multi-mode encoder is used to record various physiological parameters and covers the full range of objective physiological signals used in clinical observations and biofeedback.

### Procedure

**Fig. 4:**
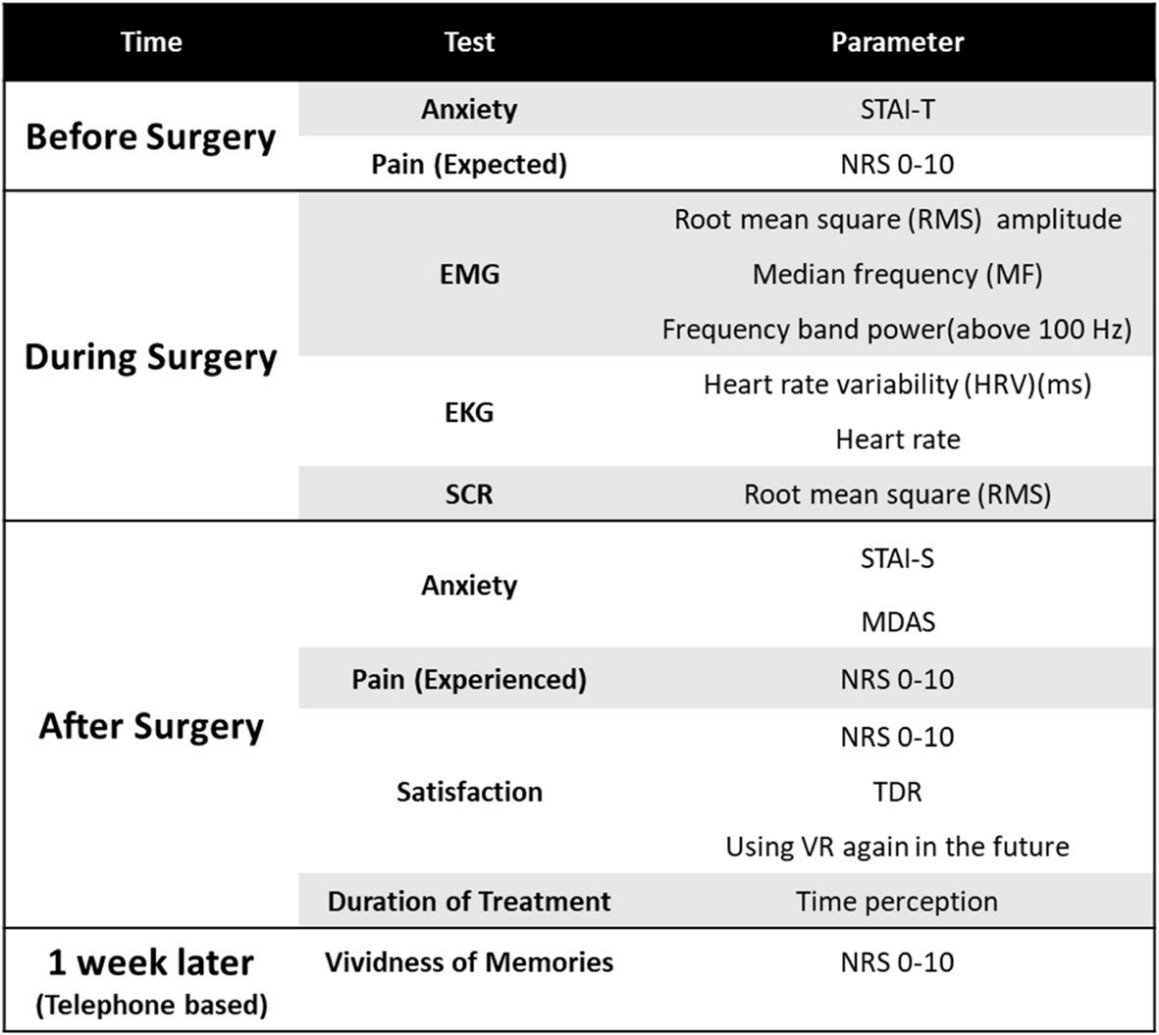
Different tests taken during study

Study participants underwent panoramic radiography (CRANEX D, Soredex, Tusula Finland) and cone-beam computed tomography (CBCT, NewTom Giano, Verona, Italy) to assess mandibular bone atrophy, perform surgical planning, and determine bone dimensions at the implant anchorage site. Seven days before surgery, patients underwent professional oral hygiene. One hour before surgery, antibiotic prophylaxis with amoxicillin + clavulanic acid 2 g was prescribed. Patients were asked to rinse their mouths with 0.2% chlorhexidine mouthwash from three days before the first surgery until two weeks after the implant surgery. Before starting the surgery, patients completed several questionnaires (Fig. 4). We attached eleven electrodes to the patient’s fingers, neck, and wrists to collect physiological information (Fig. 2). The treatment duration was 25 minutes in each process, whether with VR or without VR (excluding patient preparation time, anesthetic injection, and its onset time).

The inferior alveolar nerve block was the technique used for the lower jaw arch. Dr. XXX performed injections for all patients. Fortunately, no patients experienced sudden pain during injection due to the needle hitting the inferior alveolar nerve or other factors; otherwise, they would have been excluded from the study. WhiteSky dental implants (Bredent, Senden, Germany) were placed in the 36 and 46 tooth areas (right and left lower first molar teeth) in groups 1 and 2 patients. Dr. XXX performed all surgeries. The implants were placed according to the implant system manufacturer’s recommendations (following the recommended drill sequence at recommended speeds). Patients were instructed to rinse their mouth twice a day with 0.2% chlorhexidine aqueous solution and avoid brushing the area for two weeks after surgery. In all cases, the prescribed post-op medication was amoxicillin 500 mg every 8 hours for seven days (in cases of penicillin allergy, 300 mg of clindamycin every 8 hours was prescribed) and ibuprofen 600 mg every 8 hours for three days. Sutures were removed seven days after surgery.

### Psychometric assessment

#### Pain

**Fig. 5:**
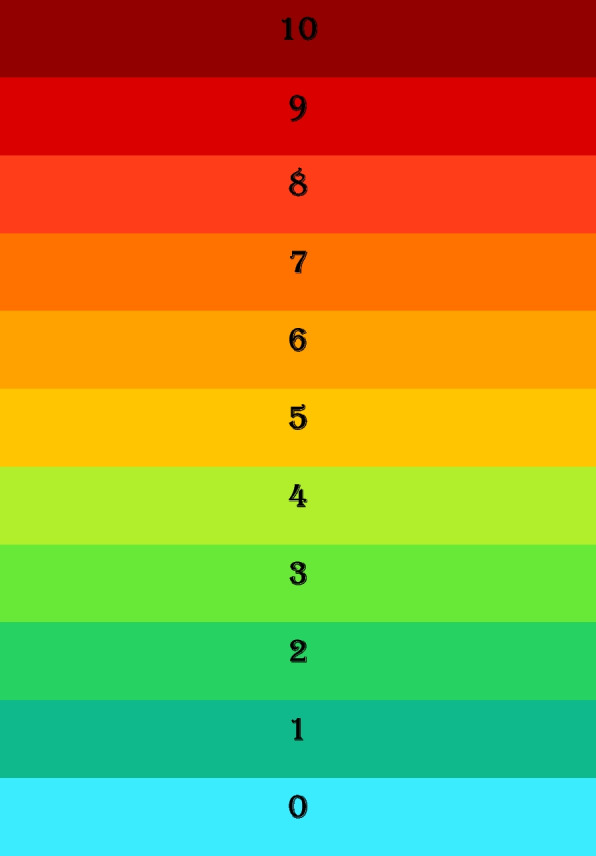
Modified 11-item NRS questionnaire. This is a common and famous test to measure a subjective variable that we modified by using different colors

A modified single-item NRS questionnaire assessed the expected or experienced pain intensity during surgery (“How would you rate the pain you will experience during the surgery,” 0 being no pain to 10 being the worst possible pain). This was administered at both surgical sessions (Fig. 5). This test is suitable because first, it shows pain intensity with different colors that can be more precise than using just numbers for comparison between patients; second, it does not use any facial expressions or phrases, so some patients do not self-censor, especially men who, for pride and other reasons, report less pain felt. At the start of the session, this test was titled “expected pain” (how much pain do you expect to have?), and 5 minutes after completing the surgical process as “experienced pain” (how much pain did you experience during surgery?). Patients were asked to mark the number of their choice with a pen.

#### Anxiety

1. STAI (State-Trait Anxiety Inventory). This questionnaire is extensively used in research and clinical activities and includes separate self-report scales for measuring state and trait anxiety [46]. Each scale consists of 20 items that can be scored from 1 (not at all) to 4 (very much). The total score for STAI-S or STAI-T ranges from 20 to 80. The first 20 questions, state anxiety (S), can be considered as a cross-section of a person’s life, meaning its occurrence is situational and specific to stressful situations (arguments, loss of social status, threats to human security and health) and shows the person’s feelings at that moment. But the next 20 questions, trait anxiety (T), refer to individual differences in response to stressful situations with varying degrees of state anxiety. Patients were told to choose only one response option for each question and leave none unanswered.
2. MDAS (modified dental anxiety scale). In addition, each patient’s anxiety level was assessed using the Modified Dental Anxiety Scale (MDAS) questionnaire. The MDAS is the most commonly used dental anxiety questionnaire in the United Kingdom and is a modified version of the DAS questionnaire, the most prevalent measurement in studies related to dental anxiety [17]. This questionnaire consists of five items to assess the anxiety level in different dental situations. Each question has a 5-point Likert scale response ranging from “not anxious” to “extremely anxious”. Each response is scored, and the sum of all responses is recorded. The total score on this scale ranges from 5 to 25. It is important to note that Humphris and Hall found that using this questionnaire did not increase anxiety [2].

#### Satisfaction

1. After the surgery, an 11-point NRS questionnaire was administered to participants to rate their satisfaction with their treatment on a scale of 0 (completely dissatisfied) to 10 (completely satisfied).
2. Patients were also asked if, given the choice next time, they would want the dentist to use the virtual reality headset again or not. Options were yes, no, and not sure. Patients had the option to leave their comments in the questionnaire feedback section.
3. Treatment Distress Rating (TDR) was evaluated after completion of treatment to assess the dentist’s distress resulting from the dental procedure for the patient. In this way, the dentist was asked to determine the amount of distress caused by VR during treatment and its interference with the surgeon’s freedom of action compared to the control group, i.e., when routine treatment (without VR) was performed for them. The response format was similar to the 11-point numerical rating scale (NRS) for pain, with 0 defined as “no distress at all” and 10 as the “worst possible distress.”

#### Vividness of memories

One week after each surgery session, patients were contacted by phone and asked to complete the telephone questionnaire. We prepared a questionnaire in the form of an 11-point NRS (from 0, meaning not at all, to 10, meaning completely remember) that assessed the vividness of experienced memories. The items were adapted from the study of Tanja-Dijkstra et al. [48] for relevance to this study’s clinical and dental context and measured with the following questions: (a) How vividly do you imagine the visit? (b) How vividly do you feel the emotions you experienced? (c) How vividly do you remember the discomfort of keeping your mouth open? (d) How vividly do you remember the sound of the dental instruments? (e) How vividly do you remember the smell in the dental office?

#### Time perception ratio

Patients are asked, “How long do you think the entire process of your treatment was from the beginning of the surgery to the end, in minutes?” It was assured that none of the patients could observe the elapsed time through a clock or other device. The sensed time amount was expressed as a fraction of the actual time elapsed for each patient.

### Psychophysiological assessment

EMG, or electromyography, is a method to detect, diagnose, and analyze the electrical signals that originate in muscles. The EMG sensor, also known as an electromyography sensor, is a sensor that measures the small electrical signals generated by muscles when they move. The active electrodes (blue and yellow) were placed flush on the sternocleidomastoid (SCM) muscle, and the inactive electrode (black) was placed on the clavicle bone surface.

EKG (ECG), or electrocardiogram, is a method to evaluate the heart’s electrical activity over a period of time using electrodes placed on the body in specific locations. Electrical impulses in the heart are what tell the heart muscles to contract. Contractions are what we refer to as heartbeats. An EKG provides a visual representation of electrical activity in the form of charted waves, and these tracings provide information about heart rate and heart rate variability. The electrodes were used with wrist straps, with the yellow electrode on the right wrist and the blue and black electrodes on the left wrist and the Radius bone, respectively.

SCR, or Skin conductance response, measures conductivity between two electrodes on the skin. A small voltage is applied to the skin to measure resistance, and the skin conductance is measured. Skin conductance depends on sweat gland activity and the size of skin pores. The basal skin conductance of individuals varies for many reasons, including gender, diet, skin type, and location. Sweat gland activity is controlled to some extent by the sympathetic nervous system. When a subject is startled or experiences anxiety, skin conductance (for a few seconds) increases due to increased sweat gland activity (unless the glands are saturated with sweat). After a stimulus, skin conductance naturally decreases due to reabsorption. In this study, SCR sensors are sewn into Velcro straps and fastened to two fingers.

### Data collection

The clinical information, all details of the surgical procedure, monitoring the completion of questionnaires, and all data obtained from each patient were recorded by the study responsible (XXX) in an electronic database.

### Statistical analysis

Data analysis in this study was performed to Treat the approach. The data analysis of the present study was conducted in two sections: Descriptive Statistics and Inferential Statistics. The descriptive statistics section reported measures of central tendency and dispersion, along with tables and charts. In the inferential statistics section, the normality of the data was examined using the Kolmogorov-Smirnov test. The independent Samples T-test was used to compare the age variable between the two groups, and the Chi-Square test was used to compare the gender distribution between the two groups. The Generalized Estimating Equations (GEE) model was used to investigate the effect of intervention type, gender, and age variables. Model assumptions were checked by residual analysis. SPSS Inc. software was used for data analysis) Released 2009. PASW Statistics for Windows, Version 18.0. Chicago: SPSS Inc. (The significance level in this study was considered 0.05.)

## Results

In the present study, there were 73 patients, of whom 36 (49.3%) were male, and 37 (50.7%) were female. The mean age of the patients was 44.92 ± 12.98 years. The Kolmogorov-Smirnov test showed that the study variables had normal distributions (*P* > 0.05). There was no statistically significant difference in mean age between the two groups (*P* = 0.830). There was no statistically significant difference in age distribution between the two groups (*P* = 0.908) (Table 1). The mean values of the study variables according to the type of intervention are presented in Table 2. The GEE model was used to investigate the effect of intervention type, gender, and age on the variables under study (Table 3).

**Table 1:** Patients’ characteristics (*n* = 73)

		Group 1 (N: 37)	Group 2 (N: 36)	Total	*P*-value
Gender	Male	18	18	36	0.908^‡^
	Female	19	18	37	
Age	Year	45.24 ± 13.03	44.58 ± 13.10	44.92 ± 12.98	0.830^†^
Education	Primary school	4	4	8	0.942^*^
	High school	16	14	30	
	University	17	18	35	

Mean ± standard deviation and count (percentage %) are presented for continuous and categorical data, respectively

^†^Independent-Samples T-Test

^‡^Chi-Square Test

^*^Chi-Square Monte Carlo

**Table 2:** Average study variables by type of intervention

	Intervention			
	Without VR		With VR	
	Mean	SD	Mean	SD
Expected Pain	5.85	.92	5.93	.89
Experienced Pain	4.30	1.32	2.59	1.09
MDAS	17.42	3.76	12.88	2.64
STAI-S	41.07	6.04	37.62	5.68
STAI-T	41.05	4.26	41.23	4.21
Satisfaction	7.64	1.19	8.78	1.20
Time Perception Ratio	1.20	.20	1.08	.20
Vividness of Memories	4.98	.87	4.29	.80
Treatment Distress Rating	3.49	1.20	6.95	1.70
Root mean square (RMS) amplitude of EMG	62.22	4.03	48.06	4.72
Median frequency (MF)	71.22	3.65	83.99	5.42
Frequency band power(above 100 Hz)	70.62	4.14	51.97	2.38
Heart rate variability (HRV)(ms)	58.27	1.08	54.52	.56
Heart rate	81.10	3.11	70.80	2.23
Root mean square (RMS) of GSR (SCR)	21.35	2.00	15.96	2.08

**Table 3:** The effect of the type of intervention, gender and age on the studied variables of the study with the GEE model

Dependent Variable	Parameter		B	Std. Error	95% Wald Confidence Interval		Hypothesis Test		
					Lower	Upper	Wald Chi-Square	df	*P*-value
Expected Pain	(Intercept)		5.481	.2789	4.935	6.028	386.278	1	< 0.001
	Sex	Female	.125	.1639	−.196	.446	.580	1	.446
		Male (ref)	0	–	–	–	–	–	–
	Age		.007	.0057	−.004	.018	1.429	1	.232
	Intervention	With VR	.082	.1296	−.172	.336	.402	1	.526
		Without VR (ref)	0	–	–	–	–	–	–
	(Scale)		.818						
Experienced Pain	(Intercept)		4.396	.3560	3.699	5.094	152.477	1	< 0.001
	Sex	Female	.231	.1842	−.130	.592	1.577	1	.209
		Male (ref)	0	–	–	–	–	–	–
	Age		−.005	.0069	−.018	.009	.464	1	.496
	Intervention	With VR	−1.712	.2109	−2.126	−1.299	65.940	1	< 0.001
		Without VR (ref)	0	–	–	–	–	–	–
	(Scale)		1.470						
MDAS	(Intercept)		15.258	1.2519	12.804	17.711	148.545	1	< 0.001
	Sex	Female	1.150	.6847	−.192	2.492	2.820	1	.093
		Male (ref)	0	–	–	–	–	–	–
	Age		.035	.0254	−.015	.085	1.925	1	.165
	Intervention	With VR	−4.548	.2642	−5.066	−4.030	296.236	1	< 0.001
		Without VR (ref)	0	–	–	–	–	–	–
	(Scale)		10.089						
STAI-S	(Intercept)		38.927	2.2344	34.547	43.306	303.494	1	< 0.001
	Sex	Female	2.158	1.3535	−.495	4.810	2.542	1	.111
		Male (ref)	0	–	–	–	–	–	–
	Age		.023	.0535	−.082	.128	.190	1	.663
	Intervention	With VR	−3.452	.2131	−3.870	−3.034	262.324	1	< 0.001
		Without VR (ref)	0	–	–	–	–	–	–
	(Scale)		33.539						
STAI-T	(Intercept)		39.793	2.0553	35.765	43.822	374.854	1	< 0.001
	Sex	Female	1.463	.9331	−.365	3.292	2.460	1	.117
		Male (ref)	0	–	–	–	–	–	–
	Age		.012	.0464	−.079	.103	.062	1	.803
	Intervention	With VR	.178	.2035	−.221	.577	.766	1	.382
		Without VR (ref)	0	–	–	–	–	–	–
	(Scale)		17.610						
Satisfaction	(Intercept)	9.140	.7308	7.708	10.572	156.438	1	< 0.001	
	Sex	Female	.019	.1780	−.330	.368	.012	1	.914
		Male (ref)	0	–	–	–	–	–	–
	Age		−.037	.0094	−.056	−.019	15.505	1	< 0.001
	Education	Primary School	.082	.4121	−.726	.889	.039	1	.843
		High School	.301	.4391	−.560	1.162	.469	1	.493
		University (ref)	0	–	–	–	–	–	–
	Intervention	With VR	1.137	.1703	.803	1.471	44.551	1	< 0.001
		Without VR (ref)	0	–	–	–	–	–	–
	(Scale)		1.194						
Time Perception Ratio	(Intercept)		1.200	.0767	1.050	1.351	245.001	1	< 0.001
	Sex	Female	.028	.0427	−.056	.112	.428	1	.513
		Male (ref)	0	–	–	–	–	–	–
	Age		.000	.0017	−.004	.003	.018	1	.893
	Intervention	With VR	−.128	.0172	−.161	−.094	55.203	1	< 0.001
		Without VR (ref)	0	–	–	–	–	–	–
	(Scale)		.040						
Vividness of Memories	(Intercept)		5.018	.3549	4.322	5.713	199.881	1	< 0.001
	Sex	Female	−.096	.1881	−.465	.273	.261	1	.609
		Male (ref)	0	–	–	–	–	–	–
	Age		.000	.0083	−.016	.016	.000	1	.984
	Intervention	With VR	−.689	.0620	−.810	−.567	123.507	1	< 0.001
		Without VR (ref)	0	–	–	–	–	–	–
	(Scale)		.706						
Treatment Distress Rating	(Intercept)		4.074	.4374	3.216	4.931	86.746	1	< 0.001
	Sex	Female	−.314	.2516	−.807	.179	1.555	1	.212
		Male (ref)	0	–	–	–	–	–	–
	Age		−.009	.0090	−.027	.008	1.094	1	.296
	Intervention	With VR	3.452	.2293	3.003	3.901	226.733	1	< 0.001
		Without VR (ref)	0	–	–	–	–	–	–
	(Scale)		2.154						
Root mean square (RMS) amplitude of EMG	(Intercept)		60.283	2.4138	55.552	65.014	623.704	1	< 0.001
	Sex	Female	−.037	1.2182	−2.424	2.351	.001	1	.976
		Male (ref)	0	–	–	–	–	–	–
	Age		.049	.0596	−.067	.166	.686	1	.408
	Intervention	With VR	−14.158	.9975	−16.113	−12.203	201.468	1	< 0.001
		Without VR (ref)	0	–	–	–	–	–	–
	(Scale)		19.742						
Median frequency (MF)	(Intercept)		69.647	2.5296	64.689	74.605	758.043	1	.000
	Sex	Female	−.534	.9449	−2.386	1.318	.320	1	.572
		Male (ref)	0	–	–	–	–	–	–
	Age		.048	.0616	−.073	.169	.611	1	.434
	Intervention	With VR	12.766	1.3545	10.111	15.420	88.824	1	.000
		Without VR (ref)	0	–	–	–	–	–	–
	(Scale)		21.891						
Frequency band power (above 100 Hz)	(Intercept)		70.433	1.9098	66.690	74.176	1360.183	1	.000
	Sex	Female	.205	.9265	−1.611	2.021	.049	1	.825
		Male (ref)	0	–	–	–	–	–	–
	Age		.001	.0468	−.090	.093	.001	1	.974
	Intervention	With VR	−18.648	.8082	−20.232	− 17.064	532.402	1	.000
		Without VR (ref)	0	–	–	–	–	–	–
	(Scale)		11.803						
Heart rate variability (HRV)(ms)	(Intercept)		58.327	.4180	57.508	59.146	19,471.727	1	.000
	Sex	Female	−.360	.2252	−.801	.081	2.556	1	.110
		Male (ref)	0	–	–	–	–	–	–
	Age		.004	.0115	−.018	.027	.142	1	.707
	Intervention	With VR	−3.752	.2120	−4.168	−3.337	313.388	1	.000
		Without VR (ref)	0	–	–	–	–	–	–
	(Scale)		.736						
Heart rate	(Intercept)		80.965	1.1371	78.736	83.194	5069.845	1	.000
	Sex	Female	.505	.5823	−.636	1.647	.753	1	.386
		Male (ref)	0	–	–	–	–	–	–
	Age		−.005	.0260	−.056	.046	.032	1	.858
	Intervention	With VR	−10.300	.7848	−11.838	−8.762	172.255	1	.000
		Without VR (ref)	0	–	–	–	–	–	–
	(Scale)		7.535						
Root mean square (RMS)of GSR	(Intercept)		20.984	1.1730	18.685	23.283	319.996	1	.000
	Sex	Female	.799	.5287	−.237	1.835	2.283	1	.131
		Male (ref)	0	–	–	–	–	–	–
	Age		−.003	.0279	−.058	.051	.015	1	.902
	Intervention	With VR	−5.389	.5116	−6.392	−4.386	110.928	1	.000
		Without VR (ref)	0	–	–	–	–	–	–
	(Scale)		4.149						

The type of intervention had no significant effect on expected pain (*P* = 0.526). Gender and age did not significantly affect expected pain (*P* = 0.446, *P* = 0.232). The type of intervention had a significant effect on experienced pain (*P* < 0.001), such that the mean experienced pain using VR was 1.712 units lower than the experienced pain without VR. Gender and age had no significant effect on the experience of pain (*P* = 0.209, *P* = 0.496).

The type of intervention had a significant effect on STAI-S (*P* < 0.001), such that the STAI-S variable with VR use was, on average, 3.452 units lower than STAI-S without VR use. Gender and age did not significantly affect the STAI-S variable (*P* = 0.111, *P* = 0.663). The type of intervention had no significant effect on STAI-T (*P* = 0.382). Gender and age did not significantly affect STAI-T (*P* = 0.117, *P* = 0.803). The type of intervention had a significant effect on MDAS (*P* < 0.001), such that the MDAS variable with VR use was, on average, 4.548 units lower than MDAS without VR use. Gender and age did not significantly affect the MDAS variable (*P* = 0.093, *P* = 0.165).

The type of intervention significantly affected patient satisfaction (*P* < 0.001), such that patient satisfaction with VR use was, on average, 1.137 units higher than patient satisfaction without VR use. Gender had no significant effect on patient satisfaction (*P* = 0.914). Age significantly affected patient satisfaction (*P* < 0.001), such that for every year increase in age, patient satisfaction decreased by 0.037 units. Education had no significant effect on patient satisfaction (*P* > 0.05).

The type of intervention had a significant effect on the vividness of memories (*P* < 0.001), such that the vividness of memories with VR use was, on average, 0.689 units lower than that without VR use. Gender and age had no significant effect on the vividness of memories (*P* = 0.609, *P* = 0.984).

The type of intervention had a significant effect on the time perceived by the patient (*P* < 0.001), such that the time perceived by the patient with VR use was, on average, 0.128 units lower than the time perceived without VR use. Gender and age had no significant effect on the time perceived by the patient (*P* = 0.513, *P* = 0.893).

The type of intervention significantly affected surgeon distress (*P* < 0.001), such that surgeon distress with VR use was, on average, 3.452 units higher than surgeon distress without VR use. Gender and age did not significantly affect surgeon distress (*P* = 0.212, *P* = 0.296).

The type of intervention had a significant effect on EMG Root Mean Square (RMS) amplitude (*P* < 0.001), such that EMG RMS amplitude with VR use was, on average, 14.158 units lower than without VR use. Gender and age did not significantly affect EMG RMS amplitude (*P* = 0.976, *P* = 0.408). The type of intervention had a significant effect on EMG Median Frequency (MF) (*P* < 0.001), such that MF with VR use was, on average, 12.766 units higher than without VR use. Gender and age did not significantly affect MF (*P* = 0.572, *P* = 0.434). The type of intervention significantly affected EMG Frequency Band Power above 100 Hz (*P* < 0.001), such that power above 100 Hz with VR use was, on average, 18.648 units lower than without VR use. Gender and age did not significantly affect power above 100 Hz (*P* = 0.825, P = 0.97).

The type of intervention had a significant effect on heart rate variability (HRV) in ms (P < 0.001), such that HRV with VR use was, on average, 3.752 units lower than without VR use. Gender and age did not significantly affect HRV (*P* = 0.110, *P* = 0.707). The type of intervention had a significant effect on heart rate (P < 0.001), such that heart rate with VR use was, on average, 10.300 units lower than without VR use. Gender and age did not significantly affect heart rate (*P* = 0.386, *P* = 0.858).

The type of intervention had a significant effect on galvanic skin response (GSR) RMS (P < 0.001), such that GSR RMS with VR use was, on average, 5.389 units lower than without VR use. Gender and age did not significantly affect GSR RMS (*P* = 0.131, *P* = 0.902).

**Fig. 6:**
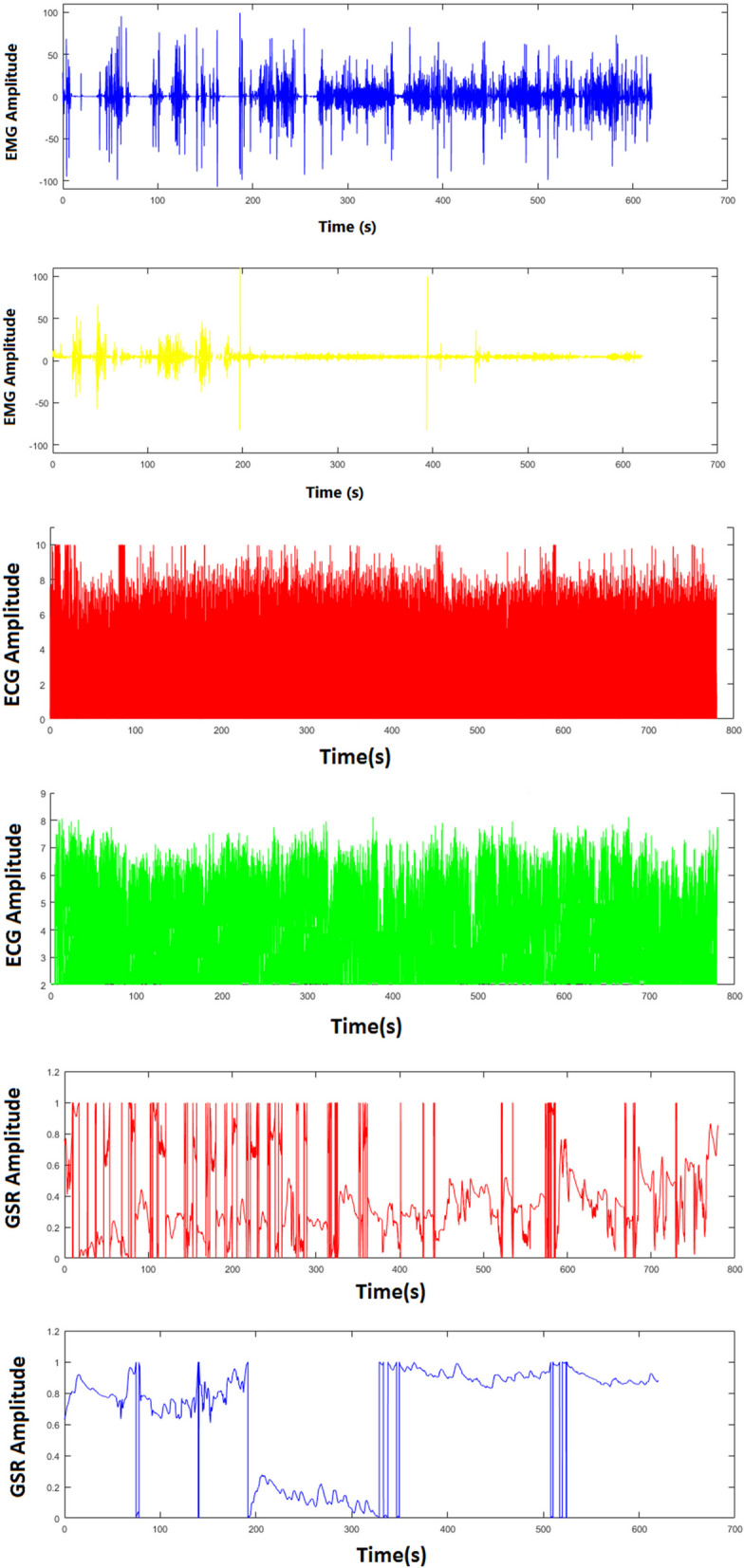
Average variables of EMG, EKG and SCR in two study groups. In each group, the bottom images are when using VR and the top image is when not using it. Positive changes are evident when patients use VR

Table 3 results indicate that younger adults found VR more useful than middle-aged and older adults. Among the six parameters that the Biofeedback & Neurofeedback device assessed (Table 4), most of the results indicated the success of VR in reducing pain and anxiety in patients during surgery. Accordingly, using VR resulted in decreased muscle tension, pain, and fatigue in the EMG parameter and decreased heart rate, sympathetic nervous system activity, pain, and anxiety in the EKG and SCR parameters (Fig. 6). The HRV variable did not support our results and its value decreased. Based on Table 5, women were less inclined than men to use VR.

**Table 4:** Biofeedback & Neurofeedback variable definitions and abbreviations

**EMG**	Root mean square (RMS) amplitude of EMG	This parameter measures the average amplitude of the EMG signal over a given time period. Increased RMS amplitude has been associated with increased muscle tension and pain.
	Median frequency (MF)	Median frequency (MF): This parameter measures the frequency at which half of the power in the EMG signal is contained. Decreased MF has been associated with increased muscle fatigue and pain.
	Frequency band power	Frequency band power: This parameter measures the power of the EMG signal in different frequency bands. Increased power in the high frequency band (above 100 Hz) has been associated with increased muscle tension and pain.
**EKG (ECG)**	Heart rate	Heart rate is the frequency between the beginning of one pulse to the beginning of the next and is expressed in a specific time window (usually one minute).
	Heart rate variability (HRV)	It describes the normal changes in heart rate from one beat to the next. HRV is closely related to emotional arousal: HRV has been found to decrease in critical situations and emotional stress (meaning the heart rate is more consistent). It has also been observed that people with more daily stress and worries have lower HRV.
**SCR (GSR)**	Root mean square (RMS) of GSR	This parameter measures the change in skin conductance in response to a stimulus, such as a painful or anxiety-provoking event. Increased SCR has been associated with increased sympathetic nervous system activity and pain or anxiety.

**Table 5:** Participant response to ‘if I was to visit the dentist again, I would want VR to be used’

Responses	Frequency	Women’s percentage
Yes	90.4% (66 participant)	48% (32 participant)
No	6.9% (5 participant)	80% (4 participant)
Unsure	2.7% (2 participant)	50% (1 participant)

**Fig. 7:**
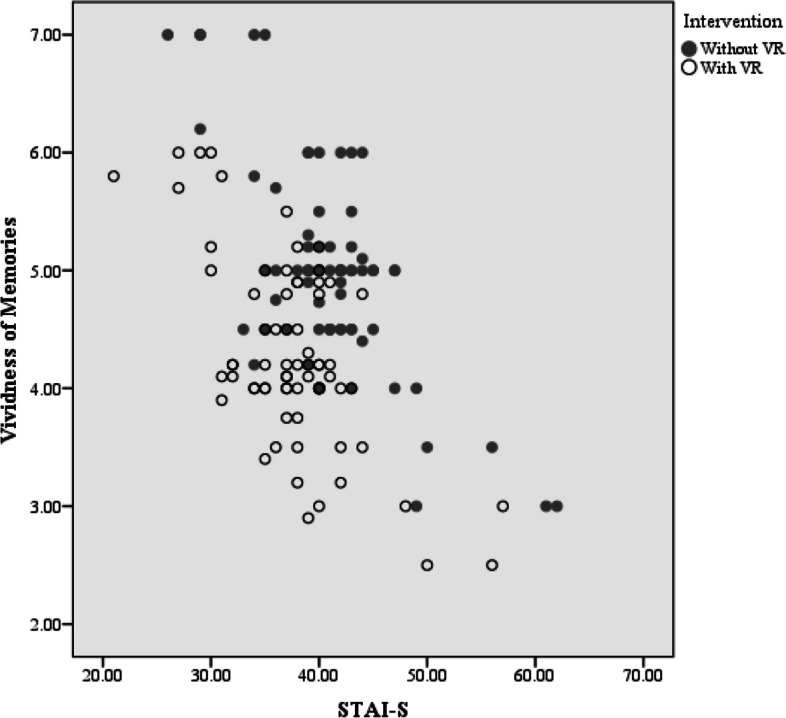
Distribution chart between STAI-S and Vividness of memories variables. There was an inverse and significant statistical relationship between STAI-S variables and Vividness of memories (Pearson Correlation, ρ = − 0.482, *P* < 0.001)

**Fig. 8:**
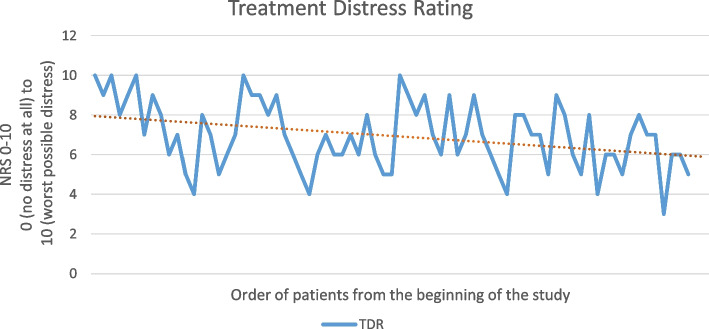
TDR diagram of the surgeon during the study, from the time of the first patient to the last. Pay attention to the downward slope of the surgeon’s scores over time

Participants with higher dental anxiety (for whom the process was likely worse) showed greater decreases in the vividness of memories compared to participants with lower dental anxiety (Fig. 7). The slope of the graph in the Fig. 8. shows that over time, the surgeon will become accustomed to using the device, with the dissatisfaction score decreasing from 9.2 for the first five patients to 5.4 for the last five patients.

## Discussion

In most studies conducted so far on the effectiveness of VR on patients, the results are not generalizable to the whole society because such studies have been biased, and participants may have different characteristics compared to others in terms of pain threshold and anxiety level. Therefore, the present study was designed as a crossover study so that each person is compared to himself or herself in two different situations. Our outcome criteria for measuring the relevant subjective and objective effects of the intervention are appropriate; the large sample size supports the robustness of the findings; and therefore, the obtained data are truly attributable to the intervention.

The present study’s results are consistent with those obtained in previous studies. Ahmadpour et al. analyzed the results of 1386 articles published between 2013 and 2019, and they found that virtual reality can be an appropriate choice for managing pain and anxiety across a wide range of medical treatments [1]. As Hoffman et al. [14] emphasized that the quality of VR equipment is important to the effectiveness of the pain reduction properties of this technique, one of the likely important reasons for patient satisfaction in our study is the use of high-resolution virtual reality headsets that are very different from other headsets used in previous articles [19]. An environment containing nature content was used to increase the impact of VR on patients. Observing natural scenery appears to reduce perceived pain by eliciting positive emotional responses and reducing stress levels [11, 33]. In the study by Tanja-Dijkstra et al. [48], two urban and coastal environments were used to reduce patients’ pain. In that study, it was interestingly shown that pain reduction was only found in the coastal environment. Even though natural elements such as trees and greenery were used to make the urban environment more effective, the results ultimately showed no difference from the control group. On this basis, it can be explicitly stated that the effect of VR is not just a distraction tool; otherwise, both environments should have had similar results. Therefore, specific VR environments that use nature have a greater effect on reducing patients’ pain.

Among the six parameters that the Biofeedback & Neurofeedback device assessed (table 4), most of the results indicated the success of VR in reducing pain and anxiety in patients during surgery. Hoffman et al. conducted a study involving functional magnetic resonance imaging brain scans, the results of which showed that the effect of VR on reducing pain was associated with a significant decrease in brain activities related to pain. VR analgesia appears to alter how signals received are processed in the brain. During the use of VR, all five regions of interest in the brain (the insula, thalamus, anterior cingulate cortex, and primary and secondary somatosensory cortex) processed fewer pain signals. These results provide additional evidence for the analgesic effectiveness of VR [13].

In this clinical trial, out of the total 73 patients studied, 12 had severe anxiety in one or both sessions (five men and seven women in total, based on the STAI-S test), of which 8 (three women and five men) reported that the use of VR was very satisfactory for them (mean satisfaction = 8), but the other 4 (all four were women) had lower satisfaction (mean satisfaction = 6.5). After completing the treatment session and questionnaires, a brief interview was conducted with these participants. One hypothesis for this difference was that in the eight people who were very satisfied with VR, the root of their anxiety was a specific object before surgery started, such as an anesthesia injection (five people) who stated after the injection they no longer had anxiety, or the overall atmosphere of the room (two people), or the word surgery (one person), but in the other four with less satisfaction, their main anxiety and fear was being vulnerable in the implant surgery process. They stated that they were unaware of what the surgeon was doing and felt more anxious with the VR headset. Therefore, as far as we can imagine, the use of VR in individuals with high stress can be very helpful [10, 47] or unhelpful [39], and this entirely depends on the patient themselves and the root of their fears. One hypothesis of the authors was that individuals with low anxiety would have less satisfaction compared to those with moderate anxiety because their anxiety level was not high enough to be greatly reduced by the effects of VR, but in practice, individuals with moderate anxiety (mean satisfaction = 8.32) had similar satisfaction to those with low anxiety (mean satisfaction = 8.34).

The present study found that anxiety levels were similar in women and men. These findings were consistent with some comparable studies that found no differences in anxiety levels between genders [7, 49]. However, some studies showed higher anxiety levels among women [6, 20, 38], and this may be because women are more likely than men to express their feelings and emotions. Additionally, it could be attributed to the fact that men refrain from reporting symptoms they consider to be weak or unmanly and tend to suffer anxiety in silence.

Based on our results, women were less inclined than men to use VR. One potential reason for this, as hypothesized in the paper by Ougradar and Ahmed [39], is their sense of claustrophobia and unawareness of their surroundings. However, given the limited number of patients studied, we cannot definitively conclude this. Some other female patients said that not being able to see the surgical tools with the VR headset made them feel more at ease during the procedure.

Our results indicate that younger adults found VR more useful than middle-aged and older adults. Interestingly, four women over 65 expressed at the end of treatment, while seated in the dental chair, that they were very satisfied with the treatment (mean satisfaction = 9.45) but no longer wanted to use VR. When asked at the end of the session why they did not want to use VR in the future despite being satisfied with it, all four of them used similar phrasing with a mocking tone, referring to VR as “ridiculous.” Their unfamiliarity with technology made them perceive it as less valuable. Therefore, younger populations seem like a better choice for VR use. However, despite the current elderly population, the elderly in the future will be far greater than the current elderly population and also much more exposed to advanced technologies. This bodes well for greater VR use in the future.

Overall, participants perspectives were positive. Most participants expressed satisfaction with how the VR system could distract them in a way that reduced their perceived anxiety and pain levels. Several patients described VR using terms like “heaven”, “fun”, “out of body experience”, and “amazing”.

Among all participants, no patient expressed discomfort at the end of VR treatment, indicating VR is at least valuable as a distraction tool and for reducing anxiety in implant surgeries. However, a small number of participants (five people) noticed that their inability to see what was happening around them made them uncomfortable. Surprisingly, some surgeons expressed similar concerns, which deterred them from participating in the study as surgeons, as they preferred to see the patients’ faces to assess their response to treatment.

In one application of a cognitive-psychological approach, we examined the role of vividness in learning about memory processing during painful experiences. The study by Tanja-Dijkstra et al. [48], which examined the same issue in routine dental treatments, found no evidence that VR reduced the vividness and intrusiveness of memories as argued by the EI (Elaborated Intrusions) theory. It could be argued this is due to the relatively low pain during routine dental treatments as dentists use local anesthetics to control pain and normal dental procedures lack high levels of anxiety and pain for most patients, but testing the EI theory in the context of dental implant surgery could have been valuable to pursue since the surgical process for implant patients involves high levels of anxiety and pain and influencing treatment memories could provide benefits for these patients. Fortunately, in dental implant surgery, we were able to impact pain memories, which is important since past memories strongly influence expectations of future experiences [21]. A recent study highlighted this key role of recall, showing that recall of previous dental appointments influences behavioral inclinations to attend future appointments [42]. Our results indicate that VR distraction during dental treatment has the potential to break the cycle of dental anxiety by preventing the formation of vivid memories.

Perhaps the biggest problem with this device at present is its bulkiness, which makes it challenging for dentists to perform treatment. Maintaining isolation is also difficult with this device. Large devices hinder the dentist’s work and restrict their freedom of action, limiting visibility of and access to the surgical site. This is why our study’s surgeon initially gave his first patient a dissatisfaction score of 10. But notably, this surgeon has been performing implant surgery for over 15 years, so such a drastic change in his 15-year process would naturally be met with dissatisfaction. Interestingly, the slope of the graph (Fig. 8) shows that over time, the surgeon will become accustomed to using the device, with the dissatisfaction score decreasing from 9.2 for the first five patients to 5.4 for the last five patients. The risks of harm from VR use are very low. The only risk that may arise from VR use is cybersickness, which manifests with symptoms similar to classical motion sickness during and after VR use. Cybersickness is rare and sometimes resolves after a few minutes of rest. Future studies should be conducted with higher quality and smaller virtual reality headsets in order to better examine the level of satisfaction of therapists and patients by removing disturbing factors such as high mass and bulky headsets. It seems that the discussion of using virtual reality headsets in treatment is similar to using old televisions at home, and the main reason for their current limited use is their bulkiness, high price, and relatively low quality, which have been solved with the advancement of technology. The future of the treatment industry will be tied to this tool.

## Conclusions

The current study supports the positive results of previous studies and emphasized the importance of “VR equipment quality” [14] and “content” [48] in reducing patients’ anxiety and pain. Our study used both already mentioned variables as highly as possible and positively affected patients. So that in addition to reducing the patient’s pain and anxiety, we were able to reduce the vividness of memories and time perception in patients. Also, the psychophysiological assessment of patients confirmed the results obtained through the questionnaires, and 90.4% of patients requested to use the headset again on their next visits.

## Data Availability

Data used to support the findings of this study are included in the article.

## Abbreviations

VR: Virtual Reality
STAI: State-Trait Anxiety Inventory
MDAS: Modified Dental Anxiety Scale
NRS: Numeric Rating Scales
TDR: Treatment Distress Rating
EMG: Electromyography
EKG (ECG): Electrocardiogram
SCR: Skin Conductance Response
GEE: Generalized Estimating Eqs
EI: Elaborated Intrusions
